# Phytotoxic and Cytotoxic Effects, Antioxidant Potentials, and Phytochemical Constituents of *Stevia rebaudiana* Leaves

**DOI:** 10.1155/2024/2200993

**Published:** 2024-06-30

**Authors:** Einstivina Nuryandani, Dedy Kurnianto, Jasmadi Jasmadi, Ardiba Rakhmi Sefrienda, Erliana Novitasari, Erni Apriyati, Yeyen Prestyaning Wanita, Siti Dewi Indrasari, Rofiq Sunaryanto, Donowati Tjokrokusumo, Alvi Yani, Indyaswan Tegar Suryaningtyas, Yusuf Andriana

**Affiliations:** ^1^ Faculty of Science and Technology Universitas Terbuka, Pontianak 78121, Indonesia; ^2^ National Research and Innovation Agency Research Center for Food Technology and Processing, Gunungkidul 55861, Indonesia; ^3^ Department of Food and Life Science Pukyong National University, Busan 48513, Republic of Korea; ^4^ Notokusumo College of Health Sciences Study Program of Pharmacy, Yogyakarta 55243, Indonesia; ^5^ National Research and Innovation Agency Research Center for Agroindustry, Cibinong 16128, Indonesia

## Abstract

Stevia (*Stevia rebaudiana*), recognized for its low-calorie, sugar-free attributes, and various health benefits, has potential applications beyond human consumption, particularly in agriculture. This study explored the potential uses of Stevia in both agricultural and healthcare contexts by examining its plant-inhibitory, cytotoxic, and antioxidant effects. The methanolic extract of Stevia leaves was fractionated into hexane, ethyl acetate, chloroform, and water fractions. These fractions were then subjected to the bioassay analyses above and underwent identification of their chemical constituents. The results indicated that the ethyl acetate fraction demonstrated significant inhibitory effects on weed germination and growth of Beggars tick (*Bidens frondosa*) (100% inhibition at 1000 ppm of dose). This fraction also exhibited the highest antioxidant activity, total phenolic, and total flavonoid contents (IC_50_ DPPH = 18.67 *μ*g/mL, 103.50 mg GAE/g fraction, and 410.16 mg QE/g fraction, respectively). In contrast, the chloroform fraction showed the highest cytotoxic effect (LC_50_ = 700.01 ppm) in the brine shrimp (*Artemia salina*) mortality evaluation. Pearson's correlation analysis revealed a positive correlation among plant inhibitory effects, antioxidant potentials, and phenolic/flavonoid contents of Stevia. FTIR spectra confirmed the presence of phenols and nonpolar components in the ethyl acetate and chloroform fractions. In addition, GC-MS analysis successfully identified Stevia's key constituents, including tetracontane, hexadecane, hexadecanoic acid, methyl ester in the ethyl acetate fraction, and spiro [4.5] decan-7-one and 6-hydroxy-4,4,7a-trimethyl-5,6,7,7a-tetrahydrobenzofuran-2(4H)-one in the chloroform fraction. This study underscores the potential of *S. rebaudiana* as a source of natural antioxidants and herbicides, offering valuable insights into its diverse applications in agriculture.

## 1. Introduction

Stevia, scientifically known as (*Stevia rebaudiana* Bertoni), is an annual plant native to Latin America and is classified under the Asteraceae family. This plant is popular as a low-sugar calorie source. The leaf of this plant contains various steviol glycosides, such as isosteviol, steviolbioside, stevioside, and rebaudioside (A–F), which are known for their sweet flavor. This plant is used as a sugar substitute in the production of sauces, jams, and dental products [1]. Using Stevia at a concentration of 5% compared to sugar achieves the same level of sweetness [2].

Stevia shows various therapeutic benefits including antihyperglycaemic, antidiarrhea, anti-inflammatory, antihypertensive, diuretic, immunomodulatory, and antitumor activities [3]. It also exhibits antibacterial and antifungal effects against *Escherichia coli*, *Bacillus cereus*, *Fusarium oxysporum*, *Lactobacillus acidophilus*, *Salmonella typhi*, and *Staphylococcus aureus* [4, 5]. The inhibitory effects of this plant against cancer, cystic fibrosis, hypertension, inflammation, obesity, and tooth decay have been reported [6]. Moreover, different chemical constituents of Stevia, such as carbohydrates, essential amino acids, fiber, and proteins [2, 7], ascorbic acid, flavonoids, and phenolic compounds which relate to some biological properties have also been revealed [8, 9].

Despite its widespread use for sweeteners and medicinal purposes, limited studies have focused on Stevia's phytotoxic and cytotoxic effects on some indicator species. A previous study investigated the cytotoxicity of Stevia against brine shrimp (*Artemia salina*) and found its methanol extract exhibited a lethal concentration (LC_50_) value of 66.78 mg/mL [10]. Another research has reported the cytotoxic properties of this herb against various cell lines, such as SKBR3, MCF-7, and MDA-MB-231. The inhibitory effects of this plant on crops have been reported in some indicator plants such as *Allium cepa* [11], *Cajanus cajan*, *Arachis hypogea*, *Glycine max*, *Cicer arietinum*, *Sorghum vulgare* [12], *Vigna mungo*, *and Triticum aestivum* [13].

Furthermore, several studies have explored the antioxidant properties of *Stevia rebaudiana* leaf. In a study conducted by Ruiz et al. [14], the antioxidant activity of two Mexican varieties of Stevia, Criolla and Morita, showed comparable DPPH free radical-scavenging activity, ranging from 86.4% to 84.3%. This activity was also reflected in its Trolox equivalent antioxidant capacity, ranging from 18.5 to 623.7 mM/mg. Escutia -Lopez et al. [15] investigated the antioxidant potential of aqueous Stevia leaf extract, demonstrating a significant inhibition of DPPH free radicals ranging from 60% to 72.37% at a concentration of 10 mg/mL. In addition, Rao et al. [16] observed a DPPH-scavenging activity of 52.46% for Stevia leaf powder at a dose of 100 *μ*g/mL. These studies collectively highlight the substantial antioxidant activity associated with *Stevia rebaudiana* leaf.

Research investigating the impact of different polarity fractions on Stevia's inhibitory effects against the growth and germination of an invasive weed like *Bidens frondosa* remains scarce. To the best of our knowledge, studies exploring how varying extraction solvent polarities influence Stevia's ability to inhibit the germination and growth of *Bidens frondosa* have not been reported. This research gap highlights the need for our current study, which aims to evaluate the inhibitory effects of Stevia against weed using different polarities of extraction solvents. By examining these phytotoxic effects, we seek to enhance our understanding of the potential use of Stevia as a natural herbicide and optimize its utilization of Stevia beyond human consumption. In addition, this study assessed the antioxidant and cytotoxic properties of this plant using the same fractions, providing insights into both its weed-inhibitory effects and antioxidant activity. This comprehensive investigation contributes valuable knowledge for agricultural and pharmaceutical applications, particularly in weed management and the development of natural product derived from Stevia.

## 2. Materials and Methods

### 2.1. Plant Materials and Reagents

The leaves of *S. rebaudiana* were purchased commercially (Indoplant Corp, Yogyakarta, Indonesia). Acetic acid, ABTS (2,2-azinobis-3-ethylbenzothiazoline-6-sulfonic acid), chloroform, aluminum chloride, Folin–Ciocalteu's reagent, ethyl acetate, hexane, methanol, DPPH (2,2-diphenyl-1-picrylhydrazyl), quercetin, potassium persulfate, and sodium acetate anhydrous were purchased from Sigma-Aldrich (Singapore, Singapore). *Lactuca sativa* seeds were purchased commercially (PT. Panah Merah, Purwakarta, Indonesia), while *Bidens frondosa* seeds were collected in Playen, Gunungkidul, Indonesia rice fields and identified by Dr. Prayoto Tonoto (https://orcid.org/0000-0002-1439-3218), a landscape ecologist. These seeds were tested for germination and more than 90% were life before being used in plant inhibitory assay.

### 2.2. Plant Fractionation

A total of 7000 g of Stevia leaves was redried at 60°C in the oven and pulverized into powder. Then, an amount of 500 g of dried leaves powder of *S. rebaudiana* was dispersed in 1000 mL of methanol and stored at room temperature for five days. After a filtration process, the filtrate of Stevia extract was evaporated by using a rotary evaporator (Buchi Rotavapor® Rotary Evaporator R3, BÜCHI Labortechnik AG, Switzerland) at 40°C and 337 mbar. Then, a total of 44.67g of the methanol extract was fractionated using different polar solvents following low to high polarity to obtain 6.17, 0.90, 0.86, and 9.83 g of hexane, chloroform, ethyl acetate, and water fractions, respectively.

### 2.3. Total Phenolic Contents (TPCs)

The Folin–Ciocalteu (FC) solution was utilized for assessing the total phenolic levels of the extracts following the study conducted by Minh et al. [17]. A volume of sample (20 *μ*L) with a concentration of 1000 *μ*g/mL was combined with 100 *μ*L FC reagent (10%). Then, an 80 *μ*L of sodium carbonate (5%) was added after giving the mixture a short twirl in a vortex. The mixture was incubated in a dark room for thirty minutes. The absorbance of the mixture was measured by utilizing a microplate reader (Multiskan GO, Thermo Fisher Scientific, Tokyo, Japan) at 750 nm wavelength. The quantities of phenolics per gram of fraction were expressed in milligrams of gallic acid equivalent (GAE).

### 2.4. Total Flavonoid Contents (TFCs)

The procedure reported by Tuyen et al. [18] was employed to elucidate the total flavonoid contents of samples. A volume of 100 *μ*L containing 1000 *μ*g/mL of sample or 10–200 *μ*g/mL quercetin was mixed with 100 *μ*L of aluminum chloride (2%). The mixture was left to incubate for 15 minutes at ambient conditions. Then, a microplate reader (Multiskan GO, Thermo Fisher Scientific, Tokyo, Japan) was used to read the absorbance at 430 nm of wavelength. The total flavonoid contents were quantified as milligrams of quercetin (QE) equivalent per gram of sample.

### 2.5. DPPH Radical-Scavenging Activity

The method reported by Andriana et al. [19] was modified to assess the antioxidant activity of Stevia by measuring the DPPH scavenging activity of the samples compared to quercetin as a positive control. A total volume of 20 *μ*L quercetin (1–20 *μ*g/mL) or sample (100–3000 *μ*g/mL) in methanol was combined with 80 *μ*L of a 0.5 mM DPPH solution. The mixture was subsequently left to incubate for 30 minutes in the dark at ambient conditions. The absorbances of samples were measured by using a microplate reader (Multiskan GO, Thermo Fisher Scientific, Tokyo, Japan) at a 517 nm wavelength. The percentage of DPPH-scavenging activity was calculated using the following equation:

(1) $$\begin{array}{c} \%\text{ DPPH scavenging activity}=\frac{A_{C}‐A_{S}}{A_{C}}\times 100\%, \end{array}$$

where *A*_*c*_ was the absorbance of DPPH without a sample, and *A*_*s*_ was the absorbance of DPPH with a sample. The antioxidant activities of all samples were expressed as inhibition concentration (IC_50_) at *μ*g/mL.

### 2.6. Phytotoxicity Assay

Plant inhibitory evaluation of Stevia was conducted using moist filter paper placed in a 12-well plate (35 mm in depth and 22.1 mm in diameter) following the method reported by Andriana et al. [20]. The seeds of the indicator plants used in this assay were lettuce (*L. sativa*) and Devil's beggar ticks (*B. frondosa*). Ten seeds of *L. sativa* and eight seeds of *B. frondosa* were planted in their respective batches of 12-well plates at room temperature. The test solution (300 *μ*L) was pipetted to each well, which had been prepared with two filter papers, and was allowed to evaporate for an hour. Subsequently, a volume of 300 *μ*L of distilled water was added to each well. The inhibitory germination, shoot, and root lengths were evaluated at five days. The plant inhibitory potentials of Stevia against *L. sativa* and *B. frondosa* were expressed as an inhibitory percentage of germination, shoot, and root size over control.

### 2.7. Brine Shrimp Lethality Assay (BSLA)

The toxicity effects of *S. rebaudiana* fractions were assessed by the brine shrimp lethality assay following the previous studies [21, 22]. A total of 1 mg of each sample was dispersed in dimethyl-sulfoxide (DMSO), and the serial dilution method was used to create solutions with various concentrations using simulated seawater (0–1000 *μ*g/mL). The mixtures were then transferred to a 12-well plate with 10 live brine shrimp larvae in 300 *μ*L of artificial seawater. The plates were examined after 24 hours to count the *Artemia* nauplii that had persisted on each plate. In this bioassay, the absence of regulated forward movement throughout the prescribed observation period of 30 seconds served as the criterion for determining mortality. The data were utilized to determine the mortality rate of brine shrimp larvae for every concentration level and the control group. Carbofuran was utilized as a positive control due to its potent toxicity and accessibility [23].

### 2.8. Identification of Chemical Functional Groups of Stevia by Fourier-Transform Infrared Spectroscopy (FTIR)

The chemical functional groups in *S. rebaudiana* were determined by FTIR analysis as outlined in a prior technique [24] to complement the GC-MS analysis. The samples were placed right away into an FTIR system (Bruker A7.8) and scanned from 490 to 4000 cm^−1^ and then every specimen was examined three times. The spectra were ATR and displayed as the percentage of transmittance.

### 2.9. Identification of Chemical Constituents of Stevia by GC-MS

The chemical constituents of Stevia fractions were determined utilizing a GC-MS system (Agilent 7890B/MSD 5977 A, Agilent Technology, Inc., California, United States) according to the previous report [25]. A 1 *μ*L sample was introduced into the HP-5MS column, an Agilent column with 30 meters long, 250 *μ*m in diameter internally, and 0.25 *μ*m thick. The split-less helium induction was employed, with GC oven temperature set to 250°C, the pressure at 7.0699 pounds per square inch, and the overall flow rate at 104 mL per minute. The starting temperature was 40°C, maintained for one minute. The temperature rate was set to increase at 10°C per minute to a maximum temperature of 325°C with a four-minute hold interval. The scanned mass range was 122 amu–1021 amu. Ions were generated via electron ionization (EI), and subsequently, positive ions were gathered in preparation for analysis via mass spectrometry (MS). Utilizing the Agilent ChemStation software, which integrated the NIST mass spectral library for data peak processing, the GC-MS system was managed.

### 2.10. Statistical Analysis

MetaboAnalyst 6.0 software (https://metaboanalyst.ca/) was utilized to analyze the data using the one-way analysis of variance (ANOVA) method. Afterward, to determine the significant difference between the control and treatment groups, a Tukey's test with a 95% confidence interval (*p* < 0.05) was conducted. Each assay was conducted three times to ensure accuracy and reliability. The data were expressed in the form of standard deviations and mean values. *Pearson's* correlation evaluation was conducted to determine the relationship between the total phenolic and total flavonoid contents of *S. rebaudiana* and its antioxidant, cytotoxicity, and phytotoxicity potentials.

## 3. Results

### 3.1. Antioxidant Potentials, TPC, and TFC

Table 1 displays the antioxidant potentials, TPC, and TFC of different fractions of *S. rebaudiana*. The DPPH free radicals and quercetin were utilized to assess the antioxidant potential of Stevia due to their significant free radical-scavenging abilities and availability. The results showed that ethyl acetate fraction demonstrated the strongest antioxidant activity (DPPH IC_50_ value of 18.67 ± 0.61 *μ*g/mL) followed by aqueous, chloroform, and hexane fractions. These findings suggest a potent ability to neutralize free radicals of the ethyl acetate fraction of Stevia. Remarkably, this fraction also exhibited the highest TPC among all the samples, measuring 103.50 ± 0.18 mg GAE/g fraction. In addition, it contained a substantial number of flavonoids, with a TFC content of 410.16 ± 0.47 mg QE/g fraction. In contrast, the hexane fraction demonstrated the greatest DPPH IC_50_ value (>1500 µg/mL), indicating the lowest antioxidant activity compared to all samples.

### 3.2. Plant Inhibitory Potential of *S. rebaudiana* Fractions

The inhibitory effects of *S. rebaudiana* on plant germination and growth were evaluated against *B. frondosa* and *L. sativa*.  Table 2 presents the results of plant inhibitory activity of *S. rebaudiana* against *L. sativa* and *B. frondosa*. The ethyl acetate fraction demonstrated a strong inhibitory activity across all parameters, followed by chloroform, water, and hexane fractions. At the dose of 1000 ppm, it showed the highest inhibition rates on *L. sativa* germination (46.67 ± 11.55%), root height (56.28 ± 6.21%), and shoot length (61.64 ± 5.90%). This fraction also completely inhibits the germination, root height, and shoot length of *B. frondosa*. Even at lower concentrations, the inhibitory effects of ethyl acetate fraction remain significant. These results show that different fractions of Stevia exhibit varying levels of plant inhibitory activity on the tested plant species.

### 3.3. Brine Shrimp Lethality Assay (BSLA)

The results of the brine shrimp lethality assay are shown in Table 3, which measured the toxicity of different Stevia fractions and included a reference compound, carbofuran. The chloroform fraction showed the highest mortality rate, LC_50_ (700.01 ± 213.62 ppm), followed by hexane (882.79 ± 262.28 ppm), ethyl acetate (1061.45 ± 46.16 ppm), and water (3391.78 ± 388.76 ppm). This fraction, at a concentration of 1000 ppm, demonstrated a mortality rate of 62.50 ± 17.68%, suggesting moderate toxicity. Similarly, the hexane extract at a concentration of 1000 ppm exhibited a mortality rate of 55.00 ± 21.21%, indicating moderate toxicity. In contrast, the water extract exhibits lower toxicity. At a concentration of 1000 ppm, it displayed a mortality rate of 15.00 ± 0.00%. These findings reveal the varying degrees of toxicity of the different fractions and reference compounds. The hexane, chloroform, and ethyl acetate fractions of Stevia demonstrate moderate toxicity with varied mortality rates and LC_50_ values. The water fraction exhibited the least cytotoxic effect, whereas carbofuran showed the highest.

### 3.4. Correlation among TPC, TFC, Antioxidant, Cytotoxicity, and Phytotoxicity Potentials of *S. rebaudiana*

**Figure 1:**
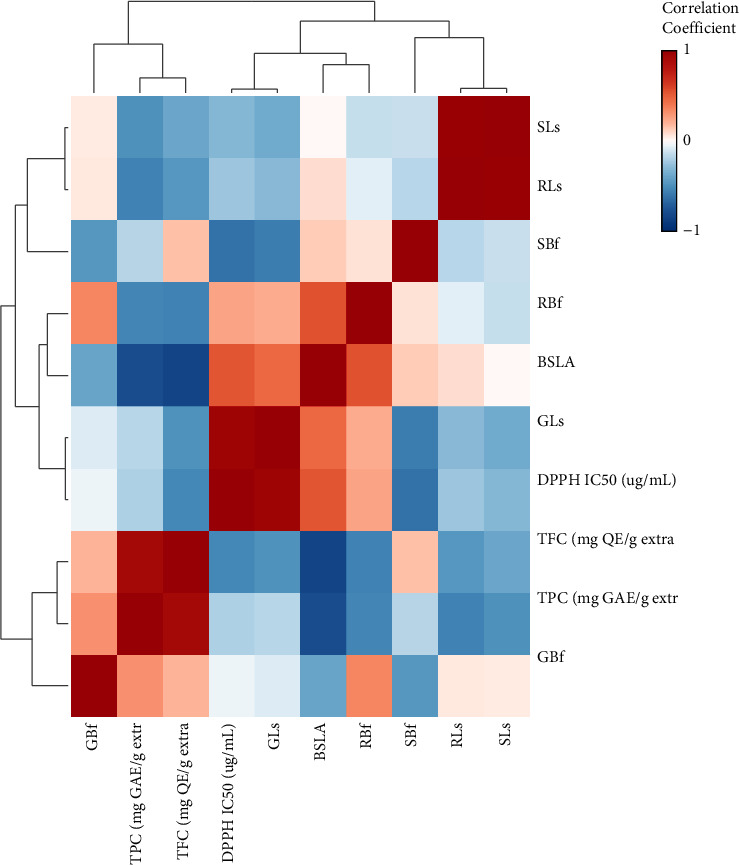
Correlation among total phenolic, total flavonoid, antioxidant, cytotoxicity, and plant inhibitory potential of *S. rebaudiana* (TFC = total flavonoid contents. TPC = total phenolic contents. IC_50_ DPPH = inhibition concentration at 50% of DPPH. LC_50_ BSLA = lethal concentration at 50% on brine shrimp lethality assay. GLs = germination inhibition at 1000 *μ*g/mL of *L. sativa.* RLs = root length inhibition at 1000 *μ*g/mL of *L. sativa.* SLs = shoot height inhibition at 1000 *μ*g/mL of *L. sativa.* GBf = germination inhibition at 1000 *μ*g/mL of *B. frondosa.* RBf = root lenght inhibition at 1000 *μ*g/mL of *B. frondosa.* SBf = shoot height inhibition at 1000 *μ*g/mL of *B. frondosa*).

Pearson's correlations of total phenolic, flavonoid, DPPH radical-scavenging activity, brine shrimp toxicity, and plant inhibitory potentials of *S. rebaudiana* are illustrated in Figure 1. Total phenolic contents showed a significantly positive correlation with *L. sativa* and *B. frondosa* growth and total flavonoid contents. These results suggested that the inhibitory effects of Stevia on *L. sativa a*nd *B. frondosa* tend to increase with rising TPC and TFC values. In contrast, TPC and TFC negatively correlated to the IC_50_ value of DPPH, which means they were proportional to the antioxidant activity of *S. rebaudiana*. Furthermore, total phenolic and flavonoid contents have a negligible correlation to brine shrimp lethality.

### 3.5. Identification of the Chemical Functional Groups by FTIR

**Figure 2:**
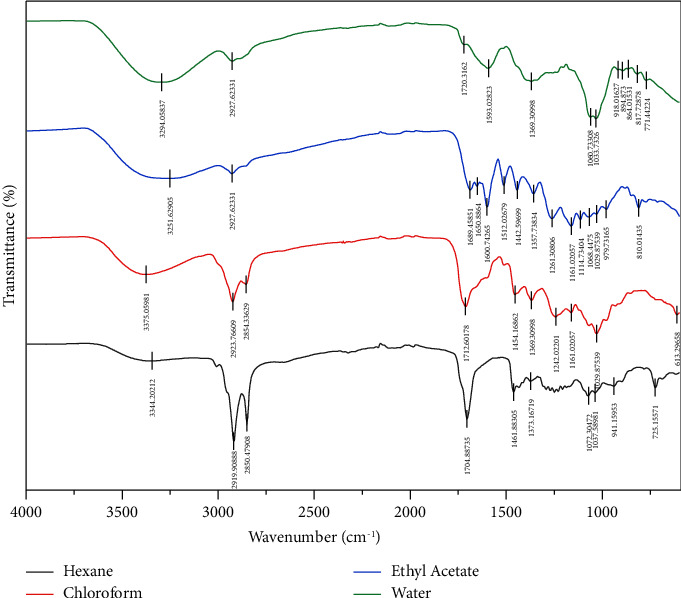
The spectra characteristic peaks associated with various functional groups in *S. rebaudiana* extracts by FTIR.

The spectra of Fourier transform infrared spectroscopy (FTIR) of fractions from *S. rebaudiana* are displayed in Figure 2. These spectra revealed peak characteristics associated with various chemical functional groups in *S. rebaudiana* fractions. The water fraction exhibited indicative peaks of O-H stretching (3294.05 cm^−1^), C-H stretching (2927.62 cm^−1^), C=O stretching (1720.31 cm^−1^), and aromatic compounds (1593.02 and 817.72 cm^−1^). The ethyl acetate fraction exhibited similar peaks, emphasizing the presence of alcohol or phenol groups (3251.62 cm^−1^), alkanes (2927.62 cm^−1^), esters (1161.02 cm^−1^), and aromatic compounds (1600.74, 1512.02, and 810.01 cm^−1^). The chloroform fraction possessed similar features to the ethyl acetate extract, with the addition of peaks indicating C=O stretching and chloroalkane groups (1242.02 cm^−1^). The hexane fraction showed a low intensity of O-H (3344.20) but was rich in alkanes (2919 and 2850 cm^−1^), carbonyl (C=O) stretch (1704), indicating the presence of aldehydes, ketones, or carboxylic acids. The hexane fraction also showed C-H bending vibration (1461) and C-O (carbon-oxygen), which is associated with alkanes, ethers, esters, or alcohols [26]. A combined FTIR with other analytical techniques is required to identify specific chemical components in the samples.

### 3.6. Identification of Phytochemical Constituents from *S. rebaudiana* Fractions by Gas Chromatography-Mass Spectrometry (GC-MS)

**Figure 3:**
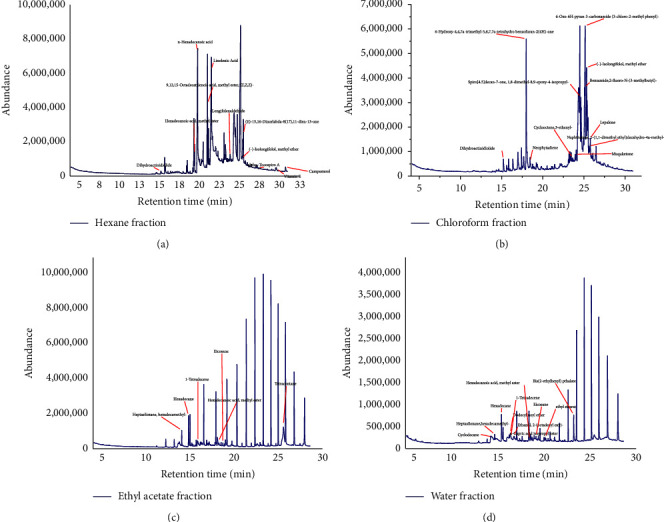
GC-MS chromatogram of (a) hexane, (b) chloroform, (c) ethyl acetate, and (d) water fractions of *S. rebaudiana.*

The phytochemical constituents of different fractions of *S. rebaudiana* are illustrated in Table 4 and the GC-MS chromatogram is in Figure 3. The GC-MS system detected 35 compounds from various chemical groups in various fractions of *S. rebaudiana*. Linolenic acid, comprising 11.91%, constituted a substantial proportion of the hexane fraction. The 6-Hydroxy-4,4,7a-trimethyl-5,6,7,7a-tetrahydrobenzofuran-2(4H)-one (6.42%) was the major component in chloroform fraction, while for ethyl acetate, hexadecane (2.58%), and hexadecanoic acid, methyl ester (1.26%) was accounted as the dominant compounds. Furthermore, in water fraction, hexadecane (3.48%) and dodecyl nonyl ether (2.27%) exhibited considerable components. These findings showed phytochemical components of *S. rebaudiana* possessed a wide spectrum of chemical groups detected by the GC-MS system.

## 4. Discussion

In this study, various fractions of *S. rebaudiana* including chloroform, hexane, water, and ethyl acetate were evaluated for their plant inhibitory, antioxidant, and cytotoxicity potentials. We found the ethyl acetate fraction exhibited the most potent radical scavenging effect against DPPH free radicals (IC_50_ = 18.67 ± 0.61 *μ*g/mL) as well as TPC (103.50 ± 0.18 mg GAE/g fraction) and TFC contents (410.16 ± 0.47 mg QE/g fraction). These findings are in line with some previous studies that evaluated ethyl acetate extracts in different species, such as *Coix lacryma-jobi* and *Tridax procumbens* [25, 27]. Generally, ethyl acetate extract contains semipolar compounds, including phenolic and flavonoids, which possess antioxidant properties [28]. Several studies also reported the correlation between TPC and TFC to antioxidant and plant inhibitory effects in some species, for example, *Anacyclus pyrethrum*, *Salvia officinalis*, and *Ambrosia trifida* [29–31]. These phenolic compounds might be responsible for the antioxidant activity of Stevia. Moreover, several studies have reported that phenolic compounds inhibit free radicals through mechanisms such as hydrogen transfer or single electron transfer via proton transfer [32]. Given the presence of phenolic compounds in the ethyl acetate fraction of Stevia, it is likely that its free radical-scavenging activity involves one or more of the following mechanisms: hydrogen atom transfer, single electron transfer, sequential proton loss electron transfer, and transition metal chelation [33]. However, further studies are needed to better understand which mechanisms contribute to the antioxidant activity of Stevia.

In line with the antioxidant activity, the ethyl acetate fraction of *S. rebaudiana* reached the highest inhibitory activity in the growth and germination of *L. sativa* and *B. frondosa* (Table 2). At the 1000 *μ*g/mL dose, this fraction suppressed the germination and growth of *B. frondosa.* It also inhibited *L. sativa* root and shoot lengths (IC_50_ shoot and root lengths = 872.78 and 764.20 *μ*g/mL, respectively) (data not shown). This inhibitory is comparable to some previous studies that reported that the ethyl acetate extract gave the most inhibitory effect on some indicator species such as *Raphanus sativus* and *Echinochloa crus-galli* compared to hexane, chloroform, and water [27, 28]. The ethyl acetate extract was reported to possess some phytotoxic allelochemicals, such as phenolic acids. For example, the ethyl acetate fraction from *Tridax procumbens* contains phenolic acids such as benzoic acid, vanillin, ferulic acid, and ellagic acid [34]. These phytochemicals have been reported to inhibit plant growth through several mechanisms, including disrupting photosynthesis and respiration, altering membrane permeability, inhibiting cell division, decreasing enzyme function and activity, and suppressing protein synthesis [35]. The current study highlights that the ethyl acetate fraction of Stevia exhibited significantly higher levels of total phenolic and flavonoid contents compared to other samples. Given the presence of these bioactive compounds, it is possible that similar mechanisms may contribute to the phytotoxic effects of Stevia. However, further analyses are needed to determine the specific modes of action of these phytochemicals in Stevia's phytotoxic activity.

In contrast with antioxidant and plant inhibitory assays, chloroform gave the most cytotoxic effect in the brine shrimp lethality assay in comparison with hexane, ethyl acetate, and water fractions. Some terpenoid compounds such as thymol, p-cymene, *γ*-terpinene, and carvacrol were reported strong toxicity in the Artemia lethality assay. These compounds induce a dose-dependent inhibition of cell proliferation, disrupt cellular processes, induce apoptosis, or interfere with cell membrane integrity [36]. This was in line with the result of GC-MS analysis that revealed chloroform fraction component consisted of benzofurans and terpenoids.

In phytochemical analysis, the GC-MS system detected the major components of ethyl acetate and chloroform fractions of *S. rebaudiana*. Tetracontane (7.10%), hexadecane (2.58%), and hexadecanoic acid methyl ester (1.26%) were counted as major compounds present in the ethyl acetate fraction. Conversely, the chloroform fraction contained dominant components such as spiro [4.5] decan-7-one, 1,8-dimethyl-8,9-epoxy-4-isopropyl (6.87%), and 6-hydroxy-4,4,7a-trimethyl-5,6,7,7a-tetrahydrobenzofuran-2(4H)-one (6.42%). Several studies reported that semipolar compounds such as ethyl acetate typically yield phenolic and flavonoid compounds which have potent of several biological activities [28, 37]. This finding underscores the importance of solvent selection in optimizing the extraction of desired phenolic substances derived from *S. rebaudiana*. However, some additional studies are required to determine the role of these compounds in the antioxidant, cytotoxic, and phytotoxic properties of *S. rebaudiana*.

## 5. Conclusions

In this study, we found that the ethyl acetate fraction of *S. rebaudiana* exhibited the most potent inhibitory effects on germination and growth of *B. frondosa* and *L. sativa*, alongside significant antioxidant activity and high total flavonoid and phenolic contents. We also revealed that the inhibitory effect on DPPH free radical scavenging, plant germination, and growth were proportional to the total phenolic and flavonoid contents of *S. rebaudiana*. Conversely, the chloroform fraction demonstrated the highest level of cytotoxicity in the brine shrimp lethality test. FTIR spectra indicated the presence of phenolic and nonpolar groups in ethyl acetate and chloroform fractions, consistent with the GC-MS, which identified several major components in the Stevia fractions. These findings suggest that Stevia has potential as a natural source of antioxidative agents and herbicides, highlighting its potential use in various sectors, including agriculture and pharmaceuticals. However, this study only evaluated Stevia's phytotoxic, cytotoxic, and antioxidant properties in a controlled laboratory environment and was limited to a few indicator species. To fully understand Stevia's potential as a natural antioxidant and herbicide and to elucidate its mechanisms of action, further research is required. This includes conducting larger-scale studies such as field trials to assess its herbicidal effects and in vivo assays to evaluate its cytotoxicity and antioxidant activity.

## Figures and Tables

**Table 1:** Antioxidant potential, TFC, and TPC of *S. rebaudiana* fractions.

Fractions	DPPH (IC_50_ (*μ*g/mL))	TPC (mg GAE/g fraction)	TFC (mg QE/g fraction)
Hexane	>1500	3.32 ± 0.18^c^	9.92 ± 0.56^c^
Chloroform	944.99 ± 7.80^a^	12.88 ± 0.12^b^	14.75 ± 0.56^b^
Ethyl acetate	18.67 ± 0.61^c^	103.50 ± 0.1^a^	410.16 ± 0.47^a^
Aqueous	641.26 ± 4.05^b^	2.88 ± 0.09^d^	4.54 ± 0.02^d^
Quercetin^*∗*^	12.06 ± 0.18^c^	ND	ND

Data are expressed in the mean ± standard deviation. According to Tukey's test, values denoted by distinct letters indicate statistically significant variations (*p* < 0.05). ^*∗*^ = positive control. ND = not determined.

**Table 2:** Inhibitory effects of *S. rebaudiana* on the germination and growth of *L. sativa* and *B. frondosa*.

Fractions	Conc. (ppm)	Inhibition (%)					
		*L. sativa*			*B. frondosa*		
		Germination	Root height	Shoot length	Germination	Root height	Shoot length
Hexane	1000	13.33 ± 5.77^cde^	43.08 ± 3.63^ab^	43.08 ± 3.63^ab^	40.63 ± 6.25^bcd^	42.88 ± 8.47^cde^	30.50 ± 10.30^c^
	500	0.00 ± 0.00^e^	39.01 ± 14.25^abc^	39.01 ± 14.25^abc^	18.75 ± 7.22^ef^	30.66 ± 2.58^def^	15.63 ± 3.14^cde^
	250	0.00 ± 0.00^e^	9.96 ± 4.43^d^	9.96 ± 4.43^d^	0.00 ± 0.00^g^	26.52 ± 4.06^f^	8.75 ± 3.75^de^

Chloroform	1000	46.67 ± 5.77^a^	29.42 ± 5.59^bcd^	29.42 ± 5.59^bcd^	53.13 ± 11.97^b^	41.29 ± 2.01^cde^	29.03 ± 7.48^cd^
	500	23.33 ± 5.77^bcd^	9.95 ± 2.02^d^	14.27 ± 5.67^cd^	34.38 ± 6.25^cde^	51.29 ± 6.29^c^	23.89 ± 12.31^cde^
	250	3.33 ± 5.77^de^	5.87 ± 1.97^d^	5.87 ± 1.97^d^	0.00 ± 0.00^g^	37.21 ± 3.33^cdef^	23.61 ± 4.05^cde^

Ethyl acetate	1000	46.67 ± 11.55^a^	56.28 ± 6.21^a^	61.64 ± 5.90^a^	100.00 ± 0.00^a^	100.00 ± 0.00^a^	100.00 ± 0.00^a^
	500	33.33 ± 11.55^abc^	29.26 ± 5.30^bcd^	29.26 ± 5.30^bcd^	47.50 ± 5.00^bc^	80.59 ± 5.77^b^	76.39 ± 7.03^b^
	250	23.33 ± 5.77^bcd^	30.56 ± 12.90^bcd^	30.56 ± 12.90^bcd^	20.83 ± 5.89^de^	44.86 ± 7.22^c^	32.92 ± 8.78^c^

Aqueous	1000	40.00 ± 10.00^ab^	24.53 ± 17.25^bcd^	24.52 ± 17.19^bcd^	21.88 ± 7.22^fg^	40.05 ± 3.20^cdef^	27.06 ± 3.90^cd^
	500	20.00 ± 10.00^bcde^	17.391 ± 6.41^cd^	15.55 ± 9.27^cd^	9.38 ± 6.25^fg^	44.59 ± 2.35^cd^	24.37 ± 9.05^cd^
	250	6.67 ± 5.77^de^	16.02 ± 2.35^cd^	11.40 ± 9.87^d^	0.00 ± 0.00^g^	29.79 ± 5.14^ef^	14.58 ± 6.25^cde^

Data are presented as the mean ± standard deviation. According to Tukey's test, distinct differences are indicated by values denoted with various letters (*p* < 0.05). Conc. = concentrations.

**Table 3:** Brine shrimp lethality test of different fractions of *S. rebaudiana*.

Fractions	Concentrations (ppm)	Mortality (%)	LC_50_ (ppm)
Hexane	1000	55.00 ± 21.21^ab^	882.79 ± 262.28^bc^
	500	37.50 ± 3.54^abcde^	
	250	30.00 ± 7.07^abcde^	

Chloroform	1000	62.50 ± 17.68^a^	700.01 ± 213.62^bc^
	500	45.00 ± 7.07^abcd^	
	250	42.50 ± 3.54^abcde^	

Ethyl acetate	1000	47.50 ± 3.54^abc^	1061.45 ± 46.16^b^
	500	22.50 ± 3.54^bcde^	
	250	12.50 ± 3.54^de^	

Water	1000	15.00 ± 0.00^cde^	3391.78 ± 388.76^a^
	500	15.00 ± 0.00^cde^	
	250	10.00 ± 7.07^de^	

Carbofuran^*∗*^	50	40.00 ± 7.07^abcde^	66.08 ± 12.61^c^
	20	20.00 ± 0.00^cde^	
	5	22.50 ± 3.54^bcde^	

Data are presented as the mean ± standard deviation. According to Tukey's test, values denoted by distinct letters imply statistically significant variations (*p* < 0.05). ^*∗*^ = positive control.

**Table 4:** The phytochemical constituents of different fractions of *S. rebaudiana.*

No	RT (min)	Compounds	Formula	MW (g/mol)	Class	Area (%)			
						Hexane	CHCl_3_	EtOAc	Water
1	14.71	Cyclodecane	C_10_H_20_	140.27	Alkanes	—	—	—	1.21
2	15.04	Heptasiloxane, hexadecamethyl-	C_16_H_48_O_6_Si_7_	533.1	Organosiloxane	—	—	0.95	1.04
3	15.28	Dihydroactinidiolide	C_11_H_16_O_2_	180.24	Benzofurans	0.25	0.78	—	—
4	15.87	Hexadecane	C_16_H_34_	226.44	Alkanes	—	—	2.58	3.48
5	16.6	n-Capric acid isopropyl ester	C_13_H_26_O_2_	214.34	Fatty acid ester	—	—	—	2.2
6	16.97	Dodecyl nonyl ether	C_21_H_44_O	312.6	Ether	—	—	—	2.27
7	17.03	1-Tetradecene	C_14_H_28_	196.37	Alkenes	—	—	0.59	0.88
8	17.4	Ethanol, 2-(octadecyl oxy)-	C_20_H_42_O_2_	314.5	Polyethylene glycols	—	—	—	1.56
9	17.99	6-Hydroxy-4,4,7a-trimethyl-5,6,7,7a-tetrahydro benzofuran-2(4H)-one	C_11_H_16_O_3_	196.24	Benzofurans	—	6.42	—	—
10	18.47	Neophytadiene	C_20_H_38_	278.5	Alkenes	0.22	0.76	—	—
11	19.39	Hexadecanoic acid, methyl ester	C_17_H_34_O_2_	270.5	FAME	1.94	—	1.26	1.58
12	19.84	n-Hexadecanoic acid	C_16_H_32_O_2_	256.42	Fatty acid	9.44	—	—	—
13	20.14	Eicosane	C_20_H_42_	282.5	Alkanes	—	—	0.24	0.94
14	21.06	9,12,15-Octadecatrienoic acid, methyl ester, (Z, Z, Z)-	C_19_H_32_O_2_	292.5	FAME	2.65	—	—	—
15	21.19	Phytol	C_20_H_40_O	296.5	Alcohol	1.84	—	—	—
16	21.3	Methyl stearate	C_19_H_38_O_2_	298.5	Fatty acid ester	0.57	—	—	0.33
17	21.54	Linolenic acid	C_18_H_30_O_2_	278.4	Fatty acid	11.91	—	—	—
18	23.66	Cyclooctene, 3-ethenyl-	C_10_H_16_	136.23	Others	0.88	0.9	—	—
19	23.87	Longifolenaldehyde	C_15_H_24_O	220.35	Others	1.46	—	—	—
20	23.95	Muquketone	C_18_H_30_O	262.4	Terpenoids	—	1.23	—	—
21	24.64	Spiro [4.5]decan-7-one, 1,8-dimethyl-8,9-epoxy-4-isopropyl-	C_15_H_24_O_2_	236.35	Terpenoids	—	6.87	—	—
22	24.79	Bis(2-ethylhexyl) phthalate	C_24_H_38_O_4_	390.6	Phthalic acids	—	—	—	2.93
23	25.08	Benzamide, 2-fluoro-N-(3-methylbutyl)-	C_12_H_16_FNO	209.26	Organofluorine	—	4.54	—	—
24	25.15	6-Oxo-6H-pyran-3-carboxamide (3-chloro-2-methyl phenyl)-	C_13_H_10_ClNO_3_	263.67	Others	—	5.98	—	—
25	25.32	(-)-Isolongifolol, methyl ether	C_16_H_28_O	236.39	Terpenes	0.67	2.48	—	—
26	25.47	(E)-15,16-Dinorlabda-8(17),11-dien-13-one	C_18_H_28_O	260.4	Terpenes	1.53	—	—	—
27	25.64	Naphthalene, 2-(1,1-dimethyl ethyl) decahydro-4a-methyl-	C_15_H_28_	208.38	PAC	—	1.65	—	—
28	25.78	Lepalone	C_10_H_12_O_2_	164.2	Ketones	—	2.96	—	—
29	27.23	. alpha.-Tocospiro A	C_29_H_50_O_4_	462.7	Terpenoid	0.2	—	—	—
30	29.6	Vitamin E	C_29_H_50_O_2_	430.7	Vitamins	0.25	—	—	—
31	27.68	Tetracontane	C_40_H_82_	563.1	Alkanes	—	—	7.1	—
32	30.76	Campesterol	C_28_H_48_O	400.7	Phytosterol	0.25	—	—	—

## Data Availability

The data used to support the findings of this study are available from the corresponding author upon reasonable request.
